# Comparison between primary closure with Limberg Flap versus open procedure in treatment of pilonidal sinus, in terms of frequency of post-operative wound infection

**DOI:** 10.12669/pjms.341.13929

**Published:** 2018

**Authors:** Muhammad Sohail Jabbar, Mahwish Mahboob Bhutta, Nayyab Puri

**Affiliations:** 1 Dr. Muhammad Sohail Jabbar FCPS Part 2 Resident, PNS Shifa Hospital, Karachi, Pakistan; 2 Dr. Mahwish Mahboob Bhutta, Medical Student, Bahria University Medical and Dental College, PNS Shifa Hospital, Karachi, Pakistan; 3 Dr. Nayyab Puri, FCPS Part 2 Resident, Benazir Bhutto Hospital, Rawalpindi, Pakistan

**Keywords:** Length of Stay, Pilonidal sinus, Surgical Wound Infection and Postoperative Complications

## Abstract

**Objective:**

Pilonidal sinus is a disorder of the sacrococcygeal region affecting younger individuals with a higher hair and weight distribution. Treatment involves the use of various surgical modalities, most of which are associated with a high rate of complications. Open procedure (OP) and Limberg Flap (LF) are two commonly performed surgical procedures for the correction of pilonidal sinus disease in our setup. The objective of our study was to compare the treatment of pilonidal sinus disease by primary closure with Limberg Flap verses Open procedure in terms of frequency of postoperative wound infection.

**Methods:**

The study is a randomized clinical trial (RCT) conducted at the department of surgery, military hospital, Rawalpindi, Pakistan. It was carried out over a period of 8 months from 16 February, 2015 to 16 September, 2015. Using consecutive non-probability sampling, a total of 60 patients were selected, 30 of whom underwent Limberg Flap procedure and the remaining 30 underwent open procedure. Postoperatively, observations for wound infection on date of discharge and then again on the various follow-up visits over the next 3 weeks. The data collected was then compared by applying the chi-square test, with p-value less than 0.05 considered statistically significant.

**Results:**

Our results showed that both primary closure with Limberg flap, and open procedure are comparable options in terms of wound infection. There was no statistical significance in the incidence of post operative infections, between the two surgeries.

**Conclusion:**

In terms of wound infection, both procedures are satisfactory surgical procedures for Pilonidal sinus disease.

## INTRODUCTION

Pilonidal sinus (PS) is a common disease of natal cleft in the sacrococcygeal region characterized by weak hair accumulation within and usually consists of one or more openings communicating by a fibrous track lined by granulation tissue. The condition, was first described by Mayo in 1833 who suggested that it was of congenital origin due to remnant of an epithelial lined tract.^1^ Now, however a widely acceptable view is that, it is acquired and caused by local trauma, poor hygiene, excessive hairiness, or presence of a deep natal cleft.^2^-^4^ Individuals, who are male, young, overweight, and hirsute or have a positive family history, have a predilection of developing this benign disease.^1^,^2^,^4^-^10^

Although many treatment modalities are described, surgery remains the mainstay of treatment and is aimed at a simplified procedure with minimal post-operative pain, minimal wound care, rapid wound healing, shorter hospital stay, early return to daily activities and low recurrence rate.^11^ While various different surgical techniques, ranging from Wide local excision to complex rotation flaps procedures have been developed, no single method is labeled as the ideal treatment.^12^ A study done on outcome of pilonidal sinus reported wound infection rate of 38.4% after open procedure and another study reported wound complication rate of 7% with Limberg Flap Procedure.^13^

In our setup, Open procedure is generally the preferred surgical procedure and this study compares it with Limberg Flap Procedure as the latter has reportedly better wound healing.^7^ Thus, the results of our study will greatly help surgeons in their future choice of surgical procedure for pilonidal sinus disease leading to a better choice of technique for this chronic disease and reduction in the economic burden and morbidity of the disease.

## METHODS

The study was conducted in the department of surgery at military hospital, Rawalpindi, from 16 February, 2015 to 16 September, 2015. Our sample size came to 60, as calculated by the WHO sample size calculator while keeping power of test at 90%, level of significance at 5% and anticipated P1 and P2 at 38.4% and 7% respectively. Our inclusion criteria included individuals between the Ages of 15 and 45 years, as the disease is more common in the age group when sex hormones affect the hair growth pattern and are known to affect the pilosebaceous glands.^8^,^9^,^14^ Also those fulfilling the diagnostic criteria of Chronic discharging sinus/sinuses in natal cleft with or without surrounding tissue inflammation and with associated pain and bleeding on clinical evaluation were also included in our study. Our exclusion criteria involved patients who were terminally ill, had Uncontrolled diabetics, were Immunocompromised and immune-suppressed patients, had acute pilonidal abscess or patients who had undergone multiple surgeries for this disease.

After obtaining permission from Hospital Ethical Committee, all patients fulfilling the inclusion criteria were included in the study after the corresponding procedure had been explained to them in the language they understood and informed written consent was obtained. Demographic information like name, age, gender and address were recorded into a pre-designed proforma. Telephone contacts of patients were also obtained to ensure follow-up.

Sampling was a consecutive (non-probability) sampling and the patients were randomly allocated into two groups, equally. The 30 patients in Group-A underwent Open surgical excision with secondary healing while the remaining 30 patients in Group-B underwent primary closure with Limberg Flap technique. Pre-anesthetic assessment of all the patients was done prior to surgery. Patients were operated in prone position under general anesthesia. And the relative procedures were performed.

### Limberg Flap Procedure

In Limberg flap procedure, the patient placed in prone and the pilonidal sinus, marked as a rhombic area, with the long axis of the rhombus aimed to include all of the diseased area. The long axis is incised to excise all of the pilonidal sinus and its extensions. While the other axes are rotated to cover the midline defect in such a way that the resultant closure is via a midline suture. A vacuum drain is placed and the skin closed with antibiotics started.

### Open procedure

Open procedure involved a wide excision of the pilonidal sinus tract and healing by secondary intention.

After the operation, dry dressing was done for 48 hours and wound was examined for any signs of surgical site infection, such as swelling, redness and discharge. Both groups of patients were given similar analgesics Subsequent dressing was done daily. Stitches were removed on 10^th^ post-operative day and patients were followed for up to three weeks. In total, patients were assessed post-operatively, on day of discharge, Day 7, 14 and 21 for the variables included in the study. The total numbers of days to discharge and subsequently return to work noted. Observational Bias was controlled by training the Observer and by using a single observer.

The data thus collected, was recorded and then entered into SPSS version 13.0 and the two groups were subsequently compared for Wound infection by applying Chi-Square test. A P-value of ≤ 0.05 was considered statistically significant.

## RESULTS

In our study, out of total 60 patients, 93.3% (n=56) patients were males and 6.7% (n=4) were females. Group-A had 90% (n=27) males and 10% (n=3) females. Group-B had 96.6% (n=29) males and 3.4% (n=1) females (Table-I). The age distribution ranged from 17-41 years in the study, with the mean age in Group-A being 28.43 (SD ±6.08) and the mean age in Group-B was 27.40 (SD ± 5.90). Minimum weight in our study was 51 kg and maximum was 95 kg. In Group-A mean weight was 68.1 (SD ±9.14) and in Group-B mean weight was 68.43 kg (SD ±7.51).

**Table-I:** Stratification of groups with regards to gender (n=60).

Gender	Groups		Total (n=60)	p-Value

	Group-A (n=30)	Group-B (n=30)		
Male	27	29	56	0.301
Female	3	1	4

In total patients, 18.3% (n=11) developed wound infection. There were 20% (n=6) patients in Group-A, who got wound infection as compared with 16.67% (n=5) patient in Group-B, during the course of study (Table-II). The groups however, did not have a statistically significant difference in the frequency of wound infection as the ‘p' value was 0.739. These results have been summarized in the following tables:

**Table-II:** Stratification of groups with regards to infection rate (n=60).

Infection	Groups		Total (n=60)	p-Value

	Group-A (n=30)	Group-B (n=30)		
No	24	25	49	0.739
Yes	6	5	11	

## DISCUSSION

The published studies so far suggest that there is still some controversy regarding the best method for treatment of pilonidal sinus disease.^7^,^11^,^13^,^15^ There is universal agreement in the published literature that on pathological basis, sacrococcygeal sinus disease is an acquired condition.^2^-^6^ There is a long list of procedures that are advocated for the treatment of chronic pilonidal sinus disease and this range from total conservative treatment and non-surgical approach, to extensive surgical procedures involving extensive full thickness flaps techniques. Despite this broad range of surgical armament, the ideal treatment of pilonidal sinus disease remains a topic of debate and controversy.^11^,^13^,^15^-^18^ The ideal surgery should be simple, aiming to remove all the sinus tract as well as the predisposing factors that contribute in the formation of the pilonidal sinus.^2^,^12^ It should result in a low recurrence rate, a short hospital stay associated with minimum pain and wound healing problems, allowing the patient to resume his routine activities as soon as possible and it should have a less economic burden on the system and patient as well.^12^ However, despite extensive research there is still no seamless surgical procedure for pilonidal sinus with respect to the results of early and late complications.^19^-^21^

There was a prominent male predominance in our study. Out of the total 60 patients 93% were male and 7% female, similar gender distributions are described in other studies.^1^-^5^ The poor representation of female patients may be due to the relatively low incidence of pilonidal sinus disease in females, as females are less hirsute, also another reason for this distribution may be contributed to the fact that female are reluctant to take medical advice and undergo surgery by male surgeons who predominate in our setup.^7^-^10^ Most patients were young with the mean age of 27.9±5.96 years. Comparable results were shown in other similar studies.^2^,^4^,^5^ Wound Infection was graded as per Southampton wound grading system.^22^ It is as follows:

**Table:** 

Grade	
0	Normal Healing
I	Normal Healing with mild bruising and erythema
II	Erythema plus other signs of inflammation
III	Clear or haemoserous discharge
IV	Major complication, Pus or deep/ severe wound infection with or without tissue breakdown, haematoma requiring aspiration.

In our study, 20% in Open Procedure And 16.67% in Limberg Flap procedure exhibited wound infections. Again similar, infection rates were shown in a local study with 25% early complication rates with Karydakis technique.^14^

Over international reports, McCallum IJ et al. conducted a systemic review and meta-analysis on all studies on pilonidal sinus, in which only Five trials (559 participants) assessed the rate of surgical site infection after open healing compared with primary closure (all techniques) and although infection rates were somewhat higher after open healing but it did not expose any statistical significance.^23^ Another study comparing open healing with Z-plasty, there was, likewise, no considerable increase in infection rates after open healing.^8^ Wound infection rates were documented to be low generally in whichever surgical technique used, one study however revealed slightly higher rates with open healing by up to 11%.^7^

Aslam MN carried out study to see the post-operative infection rates in patients who underwent Limberg flap procedure for pilonidal sinus disease. In this study he showed infection rate from 0- 20% with this technique and the recurrence rate was found to be 0-5% in the same study.^24^ In an updated version, a comparative meta-analysis of, the different techniques with primary open healing, over surgical closure, revealed, no clear benefit.

Our results are similar to international and national studies, and did not show large differences in the results between the two groups when compared for the development of wound infection.^1^,^16^,^25^-^27^ There is no significant difference between both the procedures as shown in our results with reference to wound infection (p=1.00). However, different studies used various criteria's to assess infection, pain, and patient satisfaction. In order to standardize treatment, investigations and to get results comparable worldwide, set protocols should be followed where the primary outcomes of wound healing, in terms of time, surgical site infection, and recurrence are compared.

### Limitations

The comparison was only between two methods of Pilonidal sinus surgery, other procedures were not included in study. Long term complications, like recurrence were not included in the variables, as the observational period was only 21 days post-operatively. Most patients were from Rawalpindi district and surrounding area. So the sample does not represent the entire Pakistani population. The study was carried out in a military setup and so targeted specific type of patients. The cost-effectiveness issue is also not addressed in the current study and will have to be evaluated separately.

## CONCLUSION

As the results obtained in our study, are in accordance with other local and international studies, it can be concluded that both Limberg Flap Procedure and Open procedure with secondary healing are comparable surgical options for pilonidal sinus disease treatment when studied for post-operative wound infection. The results of both procedures are almost similar, and no statistically significant difference exists between both modalities in terms of wound infections. However, other factors may be taken in account for such as recurrence rates, and effective outcomes, when deciding on the appropriate procedure to undertake.

### Author`s Contribution

**SJ**: Conceived, designed, analyzed the data, prepared and reviewed the manuscript.

**MMB:** Did literature search, prepared the manuscript and edited it.

**NP:** Prepared and Edited the manuscript.
